# Contrasting Sodium and Potassium Perturbations in the Hippocampus Indicate Potential Na^+^/K^+^-ATPase Dysfunction in Vascular Dementia

**DOI:** 10.3389/fnagi.2022.822787

**Published:** 2022-01-28

**Authors:** Sasha A. Philbert, Jingshu Xu, Melissa Scholefield, Stephanie J. Church, Richard D. Unwin, Garth J. S. Cooper

**Affiliations:** ^1^Centre for Advanced Discovery and Experimental Therapeutics, Division of Cardiovascular Sciences, School of Medical Sciences, Faculty of Biology, Medicine and Health, The University of Manchester, Manchester Academic Health Science Centre, Manchester, United Kingdom; ^2^Faculty of Science, School of Biological Sciences, University of Auckland, Auckland, New Zealand; ^3^Stoller Biomarker Discovery Centre, Division of Cancer Sciences, School of Medical Sciences, Faculty of Biology, Medicine and Health, The University of Manchester, Manchester, United Kingdom

**Keywords:** vascular dementia, neurodegeneration, metal dyshomeostasis, brain-sodium levels, brain-potassium levels, Na^+^/K^+^-exchanging ATPase

## Abstract

Vascular dementia (VaD) is thought to be the second most common cause of age-related dementia amongst the elderly. However, at present, there are no available disease-modifying therapies for VaD, probably due to insufficient understanding about the molecular basis of the disease. While the notion of metal dyshomeostasis in various age-related dementias has gained considerable attention in recent years, there remains little comparable investigation in VaD. To address this evident gap, we employed inductively coupled-plasma mass spectrometry to measure the concentrations of nine essential metals in both dry- and wet-weight hippocampal *post-mortem* tissue from cases with VaD (*n* = 10) and age-/sex-matched controls (*n* = 10). We also applied principal component analysis to compare the metallomic pattern of VaD in the hippocampus with our previous hippocampal metal datasets for Alzheimer’s disease, Huntington’s disease, Parkinson’s disease, and type-2 diabetes, which had been measured using the same methodology. We found substantive novel evidence for elevated hippocampal Na levels and Na/K ratios in both wet- and dry-weight analyses, whereas decreased K levels were present only in wet tissue. Multivariate analysis revealed no distinguishable hippocampal differences in metal-evoked patterns between these dementia-causing diseases in this study. Contrasting levels of Na and K in hippocampal VaD tissue may suggest dysfunction of the Na^+^/K^+^-exchanging ATPase (EC 7.2.2.13), possibly stemming from deficient metabolic energy (ATP) generation. These findings therefore highlight the potential diagnostic importance of cerebral sodium measurement in VaD patients.

## Introduction

Within the spectrum of age-related dementias, vascular dementia (VaD) is widely recognized as the second leading cause of dementia after Alzheimer’s disease (AD), and accounts for about 20% of all cases (Rizzi et al., 2014). VaD is characterized as a heterogeneous class of brain disorders caused by cerebrovascular pathology which results in the impairment of several cognitive domains, as well as gait disturbance (Looi and Sachdev, 1999; Bazner et al., 2000; Sachdev et al., 2004). Although VaD has received a great deal of attention in recent years, progress has been slow, primarily due to diagnostic uncertainties stemming from overlapping AD and VaD pathology (O’Brien and Thomas, 2015). Moreover, there is still no cure or effective disease-modifying treatment for VaD, due to a lack of understanding surrounding the molecular events that drive cerebrovascular pathology, and repurposed AD therapies have yielded little or no effect (Sun, 2018).

The cerebrovascular pathologies most frequently associated with VaD are arteriosclerosis, enlargement of perivascular spaces, myelin loss, and microinfarction (Deramecourt et al., 2012). Small-vessel abnormalities comprising arteriosclerosis/lipohyalinosis, commonly referred to as type-1 cerebral small-vessel disease (Pantoni, 2010), initially develop in the basal ganglia and deep white matter before expanding into the vessels of the thalamus, leptomeninges, cerebellum, and brain stem (Thal et al., 2003, 2012). Although this pattern of pathology is highly characteristic of VaD, *post-mortem* evidence suggests that AD cases also exhibit a high frequency of cerebrovascular pathology (Jellinger and Attems, 2003; Deramecourt et al., 2012), thus highlighting the pathological similarity between the two diseases. While the precise location and volume of cerebrovascular insults are known to contribute to the development of cognitive impairment in VaD, the key molecular mechanisms underlying the disease are yet to be identified.

Essential metals are vital to human physiology and are often required as cofactors for fundamental enzymatic reactions (Lothian et al., 2013). As such, the concentrations of metals outside their normal physiological ranges can lead to cellular dysfunction (Fraga, 2005). A growing body of evidence now suggests that metal dyshomeostasis is present in several neurodegenerative diseases, including: AD (Xu et al., 2017), Parkinson’s disease dementia (PDD) (Scholefield et al., 2021), and amyotrophic lateral sclerosis (Violi et al., 2020). Despite these observations, there are no available reports that have utilized similar mass-spectrometry-derived methods to investigate the metallomic status in VaD. Some studies have reported increased brain-iron levels in VaD patients compared to healthy controls using clinical imaging techniques (Liem et al., 2012; De Reuck et al., 2014; Liu et al., 2015; Moon et al., 2016; Sun et al., 2017). However, not all elements can be observed using these methods, thus limiting the scope of investigation.

Given the similarity between AD and VaD, our research hypothesis was that hippocampal-copper levels in VaD cases would be lowered to levels similar to those previously reported in AD (Xu et al., 2017). Although the hippocampus is not seen as the most severely affected brain region in VaD, the sampling of this region allowed sufficient comparison to be made with our prior metal datasets from multiple age-related dementias. Here, we used inductively coupled plasma-mass spectrometry (ICP-MS) to measure the concentrations of eight essential metals (Na, Mg, K, Ca, Mn, Fe, Cu, and Zn) and the metalloid, Se, in human *post-mortem* hippocampal tissue from cases with VaD (*n* = 10) and non-demented age- and sex-matched controls (n = 10). Increased sodium and decreased potassium levels were evident in VaD cases compared to controls; however, no evidence for cerebral copper or iron perturbations in VaD cases was observed here. These data are consistent with possible effects of energy deficiency and decreased sodium-potassium pump (Na^+^/K^+^-ATPase) activity, which could contribute to hippocampal atrophy and cognitive impairment in VaD.

## Materials and Methods

### Human Ethics

All experiments were performed in accordance with relevant UK and international guidelines and regulations as stated below. The study of *post-mortem* VaD/control tissue received local Research Ethics Committee approval (18/SW/0029) supplied by the South West Dementia Brain Bank (SWDBB), which is an NHS research ethics committee-approved tissue bank. Informed consent for the collection of tissue for the VaD/control study was obtained by the SWDBB. Consent for collection of tissue for the AD, HD, PDD and type 2 diabetes (T2D) tissue was as previously stated (Xu et al., 2017; Patassini et al., 2019; Scholefield et al., 2021) [T2D (Philbert et al. in submission)]. Experiments described in this article were not pre-registered.

### Case Selection

The inclusion criteria for VaD case selection was as follows: confirmed neuropathological diagnosis of VaD at *post-mortem* examination; a Braak stage of 3 or less; non-amyloid small-vessel disease; histopathological evidence of microinfarction; and no histopathological evidence of other neurological diseases likely to cause dementia. However, due to the limited availability of tissue, complete fulfillment of these criteria was not met by all VaD cases. All cases did have evidence of cerebral ischemic damage, a confirmed diagnosis of VaD at *post-mortem* examination (as specified by the SWDBB), and no histopathological evidence of other neurological diseases likely to cause dementia. Controls had no history of dementia and no other neurological abnormalities. While all VaD cases displayed histopathological evidence of VaD, the use of the Montreal Cognitive Assessment, which is seen as the superior neuropsychometric test for the assessment VaD (Freitas et al., 2012), was only recorded in some cases. It should also be noted that the cause of death for patient 1,008 was listed as VaD and T2D (Supplementary Table 1). Although T2D is a prominent risk factor for VaD, it can also directly lead to dementia/cognitive impairment. Group characteristics for VaD cases and controls are as shown in Table 1 and individual patient characteristics, including age and *post-mortem* delay in Supplementary Table 1. The rationale for the sample criteria used for AD, HD, PDD, and T2D are as previously stated (Xu et al., 2017; Patassini et al., 2019; Scholefield et al., 2021) [T2D (Philbert et al. in submission)].

**TABLE 1:** Group characteristics.

Variable	Control	VaD
Number	10	10
Age	82 (69–94)	84 (72–98)
Male sex, *n* (%)	4 (40)	4 (40)
*Post-mortem* delay (h)	35.7 (24–43.5)	35.1 (20–54)
Brain wt (g)	1,212 (1,032–1,480)	1,245 (1,060–1,460)
Wet-wt/dry-wt	5.67 (5.21–6.14)	5.94 (5.32–6.56)

*Values are: age, post-mortem delay and brain wt, mean (range); wet-wt/dry-wt ratio, mean (± 95% CI) averaged across all samples.*

*All differences were non-significant.*

### Acquisition and Sampling of Human Brain

Hippocampal tissue was chosen for this study on the basis of our previous analyses which identified contrasting hippocampal-copper levels in cases of AD (Xu et al., 2017) and T2D (Philbert et al. in submission), and the moderate level of neurodegeneration observed in this region for VaD. Fresh-frozen wet-weight aliquots of 50 ± 5 mg were dissected using a ceramic scalpel to avoid metal contamination. For dry-weight analysis, 50 ± 5 mg of the same sample was dissected as previously described, and dried to constant weight in a centrifugal concentrator (Savant SpeedVac™; Thermo-Fisher, Waltham, MA, United States).

### Digestion

Prior to digestion, all samples were briefly centrifuged at 2,400 × *g* (Heraeus Pico 17 Centrifuge; Thermo Fisher Scientific, Massachusetts, United States) to ensure that the tissue sat at the bottom of the tubes. Concentrated nitric acid (A509 Trace Metal Grade; Fisher, Loughborough, United Kingdom) and 5% Agilent Internal Standard mixture (5183-4681; Agilent Technologies, Cheadle, United Kingdom) was used to make the tissue digestion mixture. Calibration standards were prepared to the appropriate dilutions (Supplementary Table 2) using Environmental calibration standard mixture (Agilent 5189-4688) and 2% nitric acid digestion mix. For both wet- and dry-weight analysis, 200 mL of digestion mix was added to each sample including two empty 2 mL microcentrifuge tubes to be used as digestion blanks. All microcentrifuge tube lids were punctured using a septum remover to prevent pressure build up before being transferred into a Dri-Block DB3 heater (Techne, Staffordshire, United Kingdom) at room temperature. The temperature was set to 60°C for 30 min which was then increased to 100°C for a remaining 3.5 h. After the digestion procedure, 100 μL of each sample or digestion blank was added to 5 mL of liquid chromatography-mass spectrometry grade water in 15 mL centrifuge tubes (Greiner). Samples were retained at room temperature pending ICP-MS analysis. The experimenter was blind to sample groups during the digestion and ICP-MS analysis.

### Inductively Coupled Plasma-Mass Spectrometry

Here, we have reported both wet-weight and dry-weight values to enable direct comparisons with other publications since, in different studies of tissue metal concentrations, either may be reported. Metal concentrations were determined in the Manchester Royal Infirmary Clinical Biochemistry Department using an Agilent 7700x ICP-MS spectrometer equipped with a MicroMist nebulizer (Glass Expansion, Melbourne, Australia), a Scott double-post spray chamber and nickel sample and skimmer cones. Samples were introduced into the spray chamber using an Agilent integrated autosampler. Before each analysis, the peristaltic-pump sample tubing was replaced to limit abnormal sample delivery to the nebulizer. ICP-MS system optimization and performance reports were generated on Agilent MassHunter Workstation software (G7201A, A.01.01) prior to each analysis to ensure consistent system performance.

Scandium was used as the internal standard for all elements except Zn and Se, where germanium was used, and Mo, where indium was used. To remove spectral interferences, two collision-cell gas modes were employed. All elements were analyzed in helium mode (5.0 ml min^–1^ He), except for selenium which was analyzed in high-energy helium mode (HEHe; 10 ml min^–1^ He) following Agilent’s recommendation to reduce interference by polyatomic ion formation. Germanium and indium internal standards were analyzed in both modes. Integration times for relevant trace metals were 3 s for Se; 0.01 s for Fe; 0.03 s for Mn, Cu, and Zn; and 0.1 s for Na, Mg, K, and Ca. A multi-element method using serial dilutions of environmental calibration standards (Supplementary Table 2; Agilent 5183-4688) was implemented for each analytical batch. 50 and 5 μg/L internal standard calibration standard solutions were used as periodic quality controls 1 and 2, respectively. The limit of quantification, detection limit, and background equivalent concentrations for each trace-metal analyzed in this report were automatically generated by Agilent MassHunter software (data not shown).

### Data Analysis

Wet- and dry-weight raw ICP-MS datasets were first exported to individual Microsoft Excel worksheets where they were corrected for sample weight and dilution and then converted to units of mmol/kg or μmol/kg as appropriate. The means (± 95% CI) were calculated and the significance of inter-group differences were determined by Mann–Whitney *U* tests, due to the limited sample size (*n* = 10/group), although the majority of the metals analyzed here were found to be normally distributed using the Shapiro–Wilk test (Ca, Mg, and Se were not normally distributed). Statistical analyses were also performed on log-transformed datasets using Welch’s *t*-test following demonstration that the data were generally consistent with parametric analysis (Tables 2, 3). Means (± 95% CI) were then back-transformed to reflect the actual elemental concentrations. Results from the non-parametric analyses have been included first in the Discussion section, followed by the results of the parametric analyses.

**TABLE 2:** Wet-weight metal concentrations in hippocampus of VaD and control brains.

Element	Concentration unit	Reference isotope	Control	VaD	Mann–Whitney *U p-*value	Welch’s *t* test *p*-value*
Na	mmol/kg wet weight	^23^Na	78 (72–85)	97 (87–107)	0.0015	0.0018
Mg	mmol/kg wet weight	^24^Mg	4.82 (4.56–5.08)	4.71 (4.36–5.07)	ns	ns
K	mmol/kg wet weight	^39^K	56 (51–61)	47 (42–52)	0.012	0.0089
Ca	mmol/kg wet weight	^44^Ca	1.90 (1.52–2.28)	5.11 (–1.46–11.68)	ns	ns
Mn	μmol/kg wet weight	^55^Mn	4.97 (4.35–5.59)	5.84 (3.06–8.62)	ns	ns
Fe	mmol/kg wet weight	^56^Fe	0.72 (0.61–0.83)	0.64 (0.51–0.78)	ns	ns
Cu	μmol/kg wet weight	^63^Cu	55 (42–68)	49 (42–56)	ns	ns
Zn	μmol/kg wet weight	^66^Zn	242 (220–263)	244 (218–269)	ns	ns
Se	μmol/kg wet weight	^78^Se	1.74 (1.58–1.91)	2.22 (0.90–3.54)	ns	ns

*Data are means (± 95% CI); p-values for significance of between-group differences were calculated by Mann–Whitney U and Welch’s t test based on wet-weight measurements from control (n = 10) and VaD (n = 10) brains.*

*Data were log^10^ transformed prior to parametric analysis and then back transformed to reflect the actual elemental concentrations.

**TABLE 3:** Dry-weight metal concentrations in hippocampus of VaD and control brains.

Element	Concentration unit	Reference isotope	Control	VaD	Mann–Whitney *U p-*value	Welch’s *t* test *p*-value*
Na	mmol/kg wet weight	^23^Na	471 (403–538)	609 (495–722)	0.035	0.024
Mg	mmol/kg wet weight	^24^Mg	28 (27–29)	29 (28–31)	Ns	ns
K	mmol/kg wet weight	^39^K	321 (291–351)	304 (273–336)	Ns	ns
Ca	mmol/kg wet weight	^44^Ca	11.3 (9.1–13.4)	16.4 (7.0–25.8)	Ns	ns
Mn	μmol/kg wet weight	^55^Mn	28 (24–31)	29 (23–36)	Ns	ns
Fe	mmol/kg wet weight	^56^Fe	4.10 (3.47–4.73)	4.25 (3.67–4.84)	Ns	ns
Cu	μmol/kg wet weight	^63^Cu	276 (241–318)	334 (289–379)	0.052	0.0497
Zn	μmol/kg wet weight	^66^Zn	1,278 (1,122–1,433)	1,514 (1,322–1,705)	0.089	0.0498
Se	μmol/kg wet weight	^78^Se	9.81 (9.11–10.51)	12.27 (7.49–17.05)	Ns	ns

*Data are means (± 95% CI); p-values for significance of between-group differences were calculated by Mann–Whitney U and Welch’s t test based on non-log transformed dry-weight measurements from control (n = 10) and VaD (n = 10) brains.*

*Data were log^10^ transformed prior to parametric analysis and then back transformed to reflect the actual elemental concentrations.

Statistical calculations were performed using GraphPad Prism v8.1.1 (GraphPad; La Jolla, CA, United States). *p*-values < 0.05 were considered significant and *p*-values < 0.10 have also been tabulated. Sample-size estimates were based on observations from our previous studies, where *n* = 9 for each group was sufficient for ICP-MS analysis using human *post-mortem* brain tissue (Xu et al., 2017; Scholefield et al., 2021). *Post hoc* statistical power and sample-size estimates for both dry- and wet-weight hippocampal tissue were also calculated using G*Power v3.1.9.4 (Faul et al., 2007).

To identify possible metallomic cluster separation between VaD and our previous T2D (Philbert et al. in submission) and age-related dementia dry-weight datasets (Xu et al., 2017; Patassini et al., 2019; Scholefield et al., 2021), principal component analysis (PCA) was performed using the R-platform MetaboAnalyst (Chong et al., 2019). Pairwise PCA comparisons were also performed to see if any disease-specific metallomic discriminations existed between VaD and any of the other dementia-causing diseases measured in this study. To assess the suitability of each dataset for PCA, the Kaiser-Meyer-Olkin measure for sampling accuracy and Bartlett’s test of adequacy were first performed using SPSS version 23 (IBM; Armonk, NY). All metal datasets used for PCA were mean-centered and scaled to correct for the use of different units of measurements.

## Results

ICP-MS was used to assess eight essential metals: Na, Mg, K, Ca, Mn, Fe, Cu, Zn, and the metalloid, Se, in human fresh-frozen *post-mortem* hippocampal tissue from 10 cases with VaD and 10 age-/sex-matched controls to gain an understanding of the metallomic differences in VaD. No significant case-control differences were observed for: age, *post-mortem* delay (PMD), brain weight, or dry-weight/wet-weight tissue ratios between control and VaD hippocampal tissue (Table 1 and Supplementary Table 3).

While all cases displayed histopathological evidence of VaD without the presence of other age-related dementing diseases, one case was also diagnosed with T2D (case 1,008), which is linked to the development of dementia/cognitive impairment (Xue et al., 2019). Despite the considerable efforts made to eliminate all other possible causes of dementia amongst the VaD group, the limited availability of hippocampal tissue precluded complete fulfilment of the specified exclusion criteria. However, upon visual inspection of both wet- and dry-weight ICP-MS datasets, case 1,008 did not appear to present outlying values for any of the metals analyzed in this study.

### Na and K Perturbations in VaD Hippocampal Tissue

**FIGURE 1:**
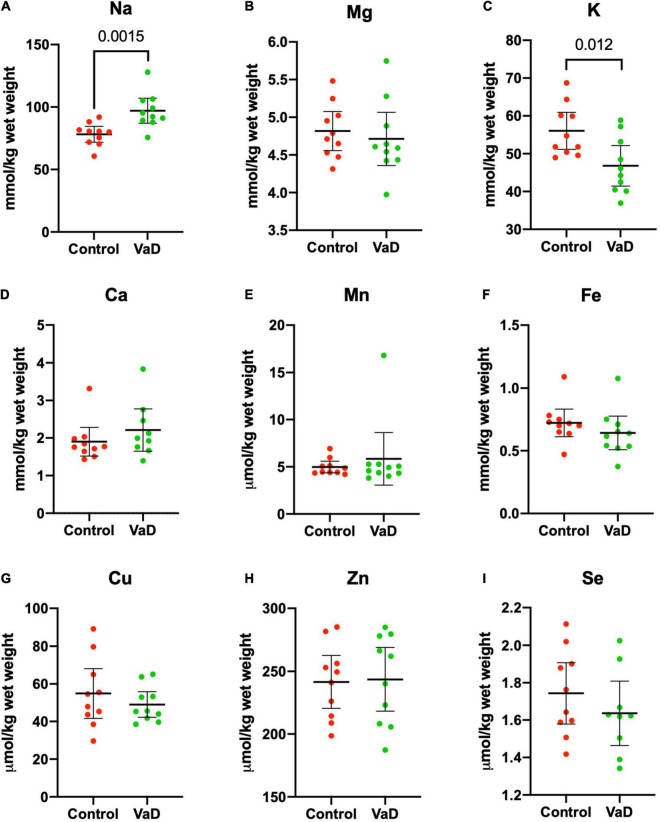
Wet-weight concentrations of nine essential elements **(A–I)** in the hippocampus compared between control (red) and VaD (green) *post-mortem* human tissue. Data shown represent elemental means ± 95% CI from control (*n* = 10) and VaD (*n* = 10) brain. A single outlier from each of Ca and Se datasets was removed from the plot for clarity. *n* = number of *post-mortem* human samples.

**FIGURE 2:**
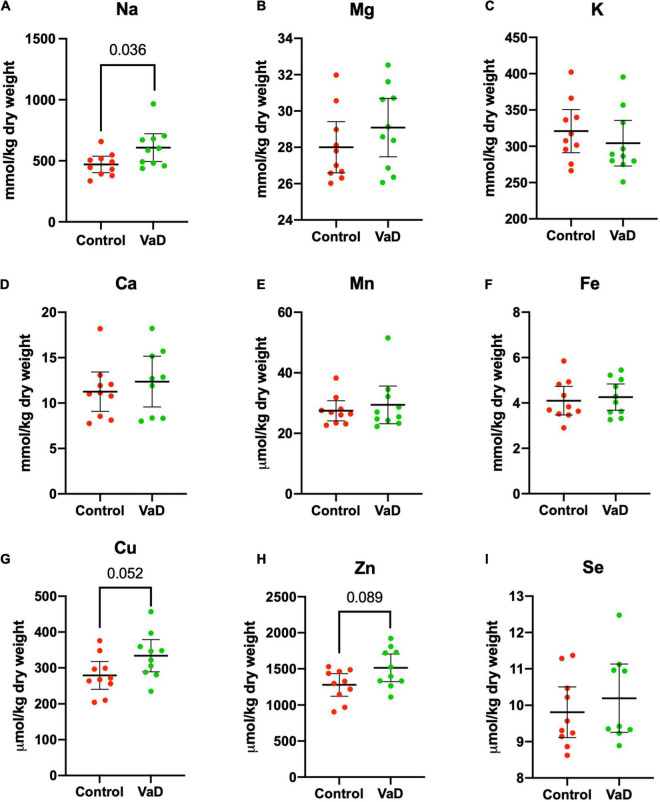
Dry-weight concentrations of nine essential elements **(A–I)** in the hippocampus compared between control (red) and VaD (green) *post-mortem* human tissue. Data shown represent elemental means ± 95% CI from control (*n* = 10) and VaD (*n* = 10). A single outlier from each of Ca and Se datasets was removed from the plot for clarity. *n* = number of *post-mortem* human samples.

In wet-weight hippocampal tissue, Mann–Whitney *U* analysis revealed robust evidence for increased Na (*p* = 0.0015) and robust evidence for decreased K (*p* = 0.012) in VaD cases (Figure 1 and Table 2), whereas, in dry-weight tissue, only moderate evidence for increased Na (*p* = 0.035) was observed in VaD cases. In addition, differences in Cu and Zn failed to reach our criteria for statistical significance (*p* = 0.052 and *p* = 0.089, respectively) (Figure 2 and Table 3).

Of the metals that were perturbed in hippocampal tissue, females displayed higher mean-metal levels for Na only in wet-weight tissue.

It has been suggested that it is harder to infer normality when using a limited sample size, hence the rationale for the use of non-parametric analysis. However, here the Shapiro–Wilk test of normality confirmed that 6/9 metals were normally distributed (whereas Ca, Mn, and Se were not normally distributed). Therefore, we also performed independent Welch’s *t*-tests on both dry- and wet-weight non-log- and log-transformed metal datasets (s). When using this parametric analysis, robust evidence for increased Na (*p* = 0.0018) and decreased K (*p* = 0.0089) in wet-weight tissue remained; however, in the dry-weight tissue, weak evidence for Cu (*p* = 0.0497) and Zn (*p* = 0.0498) perturbations, as well as increased Na levels (*p* = 0.024) were also evident (Tables 2, 3).

No other physiological metals from both sets of analyses in the present study showed any significant case-control differences.

To further understand the significance of the Na and K perturbations in VaD, Na/K ratios were calculated for both wet- and dry-weight hippocampal tissue. In both tissue conditions, mean Na/K ratios were higher in VaD cases (wet-wt = 2.15; dry-wt = 2.03) compared to controls (wet-wt = 1.43; dry-wt = 1.49; Table 4). In our previous AD hippocampal metal dataset (Xu et al., 2017), the mean Na/K ratio was also higher in AD tissue compared to controls (Table 4). In comparison to the present study, both AD and control ratios were lower; although, the Na/K-fold change (fc) in AD (fc = 1.60) was marginally higher to those seen in wet-(fc = 1.51) and dry-weight (fc = 1.36) VaD tissue (data not shown).

**TABLE 4:** Na/K ratio comparisons between VaD and AD.

Class	Na (mmol/kg)	K (mmol/kg)	Na/K ratio	*p*-value
**Dry-weight hippocampal tissue in VaD, this study**				
Control	471 (403–538)	321 (291–351)	1.49	0.031
VaD	609 (495–722)	304 (273–336)	2.03	
**Wet-weight hippocampal tissue in VaD, this study**				
Control	78 (72–85)	56 (51–61)	1.43	0.005
VaD	97 (87–107)	47 (42–52)	2.15	
**Dry-weight hippocampal tissue in AD, Xu et al. (2017)**				
Control	389 (321–458)	323 (301–345)	1.2	0.001
AD	595 (525–664)	321 (263–379)	1.92	

*Data are means (± 95% CI); p-values for significance of between-group differences were calculated by Welch’s t-tests based on measurements from control (n = 10) and VaD (n = 10)/control (n = 8) and AD (n = 9) brains.*

Retrospective statistical power analysis using an 80% minimum power requirement revealed that wet-weight Na (93.6%), but not wet-weight K (79.7%), fulfilled this criterion (Supplementary Tables 4, 5).

### No Distinguishable Separation in Hippocampal Metals Between Dementia-Causing Diseases in This Study

**FIGURE 3:**
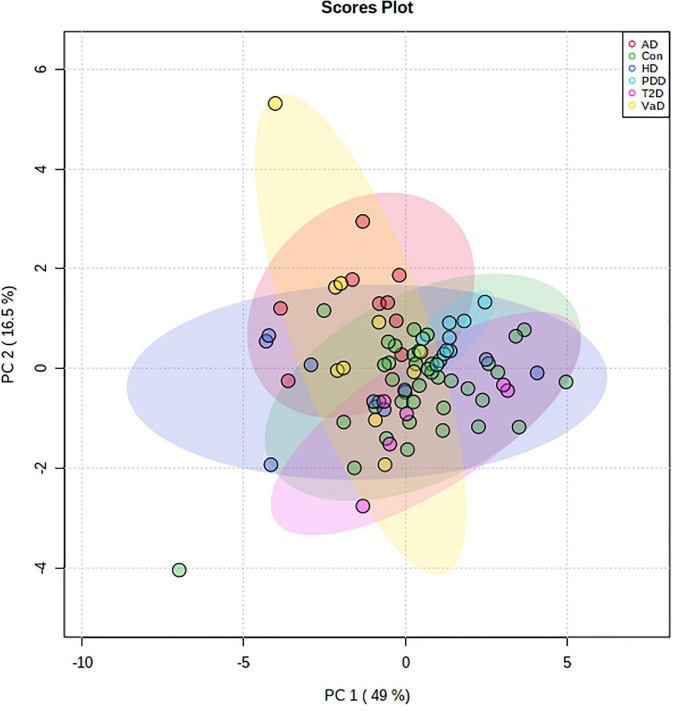
Two-dimensional PCA plot for human dry-weight hippocampal *post-mortem* tissue from multiple age-related demented diseases and T2D. Data represents a PCA plot using ICP-MS-metallomic data from VaD (*n* = 10; yellow), AD (*n* = 9; red), HD (*n* = 9; purple), PDD (*n* = 9; blue), T2D (*n* = 6; pink), and control (*n* = 40; green) dry-weight hippocampal *post-mortem* tissue. The colored ellipses around each cohort signify 95% confidence regions. The first (Dim1) and second (Dim2) principal components contribute to 49 and 16.5% of the total variance, respectively. No visible separation is apparent between all cohorts. *n* = number of *post-mortem* human samples. AD, Alzheimer’s disease; Con, Control; ICP-MS, Inductively coupled plasma-mass spectrometry; PDD, Parkinson’s disease dementia; T2D, Type-2 diabetes; VaD, Vascular dementia.

Given the evidence of perturbed hippocampal Na and K levels in VaD, we used PCA to further understand these differences in relation to our previous age-related-demented disease and T2D datasets, which were analyzed using the same methodology. As control groups from all cohorts displayed substantial overlap, all controls were merged and thereafter used as a single control cohort for comparison against all cases (Supplementary Figure 1). In the combined control PCA cohort, two controls from the PDD dataset were removed as a result of missing values, and two controls from the T2D dataset were removed because of extreme Ca outliers. In total, dry-weight hippocampal *post-mortem* tissue from four age-related dementias, including: VaD (*n* = 10), AD (*n* = 9), HD (*n* = 9), and PDD (*n* = 9); as well as T2D (*n* = 6; averaged across two runs) and grouped controls (*n* = 40) were included in the initial PCA. Prior to PCA, the suitability of the analytical methods was assessed. The Kaiser-Meyer-Olkin measure for sampling accuracy for the hippocampal dataset was 0.801, which provides robust evidence for the adequacy of these datasets for PCA. Bartlett’s test of sphericity was statistically significant [*X*^2^(36) = 425.902, *p* < 0.001], providing strong evidence for significant correlation between variables to support data reduction. Visual examination of the scree plot for the first nine principal components (PCs) justified the use of PC1 and PC2, which together explained 65.5% of the total variance within the dataset (Supplementary Figure 2). PCA revealed no separation between any group of cases and controls (Figure 3), thus suggesting no differences in the metallomic profile of *post-mortem* hippocampal tissue, across all neurological disorders analyzed by these methods in this study. The loading scores, which illustrate the relative contribution of each element to the PCs, indicated that Mg (−0.443) and Zn (−0.398) for PC1 and Ca (0.470) and K (−0.428) for PC2 were the highest (Supplementary Table 6). Although these metals exhibited the top two highest loadings for each PC, the respective correlation coefficients represent only a moderate relationship.

Lastly, to better understand disease specific relationships, pairwise PCA comparisons between VaD and the other dementia-causing diseases were made; however, no visible separation was apparent across all pairwise disease comparisons (Supplementary Figure 3). While all pairwise comparisons were well suited for data reduction, as measured by Bartlett’s test of sphericity (*p* < 0.001 for all datasets), the Kaiser-Meyer-Olkin measure for sampling accuracy revealed that the VaD vs AD comparison had a score of 0.404, thus suggesting its inadequacy for factor analysis. All other comparisons scored > 0.6 using the Kaiser-Meyer-Olkin measure for sampling accuracy.

## Discussion

Although VaD is widely viewed as the second leading cause of dementia amongst the elderly, Rizzi et al. (2014) available therapies have yielded little effect due to a lack of understanding about the molecular events that drive cerebrovascular pathology in VaD. Several age-related dementias have shown evidence of brain-metal dyshomeostasis (Ahmed and Santosh, 2010; Xu et al., 2017; Violi et al., 2020): these findings give reason to believe that such metal perturbations may play a role in neurodegenerative processes. However, to our knowledge, comparable metal-related analysis has yet to be reported on VaD *post-mortem* tissue.

Here we report measurements of nine physiologically essential metals in *post-mortem* hippocampal tissue from 10 VaD cases and 10 matched controls. No differences in PMD, brain weight, wet-weight/dry-weight ratio, or age were identified between VaD cases and controls in the present study. Therefore, we determined that case-control differences in brain-metal concentrations were unlikely to be affected by tissue variability in this study. In addition, although the duration of PMD is suggested to affect protein and metabolite expression, based on our group’s previous findings, it is unlikely that the PMDs presented in this study significantly altered brain-metal levels (Scholefield et al., 2020).

The maintenance of normal intracellular Na and K concentrations is critical to the generation of the membrane electrical potential and inhibition of apoptosis (Bortner and Cidlowski, 2003). Here, we observed marked elevations of Na in both wet and dry VaD tissue, as well as decreased K levels in wet VaD tissue. To the authors’ knowledge, there are no prior published reports of Na or K measurements in human hippocampal VaD brain tissue. Thus, these results represent novel insights into the molecular mechanisms that occur in VaD. These data also mirror our previous case-control investigation of Na levels in AD hippocampal tissue (Xu et al., 2017), wherein Na levels were similar (mean of 595 mmol/kg dry-wt) compared with those measured here in VaD (609 mmol/kg dry-wt).

Roughly 20% of overall ATP production is utilized by the CNS, primarily through the hydrolysis of ATP *via* the mitochondrial Na^+^/K^+^-ATPase to control intracellular Na and K homeostasis (Harris and Attwell, 2012). In AD and VaD, there is clinical evidence of impaired cerebral-glucose metabolism (hypometabolism), which is crucial for the initial stages of cellular respiration (Kerrouche et al., 2006; Mosconi et al., 2008; Seo et al., 2009). This may partly explain the elevation of hippocampal Na concentrations in VaD due to diminished ATP levels and subsequently reduced Na^+^/K^+^-ATPase activity. However, hippocampal hypometabolism has only been documented in AD, but not VaD patients, in which the primary regions shown to exhibit hypometabolism are the temporal gyrus, cingulate gyrus, and cerebellum (Kerrouche et al., 2006; Seo et al., 2009; Sanabria-Diaz et al., 2013). One explanation for this may be due to the sampling of *post-mortem* tissue. The analysis of *post-mortem* tissue, as used in the present study, represents end-stage disease, whereby measurement of hypometabolism in VaD patients utilizes clinical imaging methods, and thus possibly an earlier disease stage by comparison. Therefore, despite the absence of clinical evidence for hippocampal hypometabolism in VaD patients, increased Na levels in VaD *post-mortem* tissue may imply hippocampal energy dysfunction in late-stage VaD. This observation is consistent with proteomic findings from the middle-temporal gyrus in VaD *post-mortem tissue*, whereby downregulation of ATP synthase (electron transport chain complex V; EC 7.1.2.2) subunits (Datta et al., 2014; Wang et al., 2015) and upregulation of Na/K-ATPase alpha subunit 3 (ATP1A3) (Adav et al., 2014) were identified. Elevated Na levels may also highlight the potential application of clinical ^23^Na magnetic resonance imaging as a diagnostic method in VaD, which has already been shown to be informative in patients with multiple sclerosis (Inglese et al., 2010; Eisele et al., 2016).

Na^+^/K^+^-ATPase has also been shown to induce the development of reactive oxygen species *via* a Na/K-ATPase/Src signaling cascade (Srikanthan et al., 2016). Subsequent increases in reactive oxygen species can oxidize and therefore inhibit Na/K-ATPase subunits (Xie et al., 1990), which could contribute to the ion imbalances observed here. Although oxidative stress is well documented in VaD (Bennett et al., 2009), whether or not this signaling cascade is involved in the aforementioned ion imbalances requires further investigation.

Given the role of Na^+^/K^+^-ATPase in maintaining both Na and K homeostasis, it would also be reasonable to expect similar evidence for hippocampal-K dyshomeostasis. Indeed, in wet-weight hippocampal tissue, this is exactly what we found, as K levels were markedly decreased in VaD cases. Contrasting levels of both Na and K further imply the dysfunction of hippocampal Na^+^/K^+^-ATPase through the inability to regulate the entry of K^+^ and exit of Na^+^ from cells in VaD. Although mean dry-weight K levels in VaD were lower than controls, the difference between the two groups was not statistically significant in this case. The lack of evidence for K perturbations in the hippocampus mirrors our previous findings in dry-weight hippocampal AD tissue, where decreased K levels were instead observed in the cingulate gyrus and cerebellum (Xu et al., 2017).

Given the lack of available hippocampal VaD tissue at our disposal, we could not perform corresponding Western blot analysis of Na^+^/K^+^-ATPase. However, while the essential metals measured here have been shown to be stable for up to 72 h PMD (Scholefield et al., 2020), it is possible that the PMDs in the present study may affect Na^+^/K^+^-ATPase activity. Therefore, future studies may benefit from using extremely short PMD human tissue to measure Na^+^/K^+^-ATPase expression and activity.

As both Na and K perturbations were shown to be present in VaD cases, Na/K ratios were calculated to further understand the relevance of these findings. Several reports have shown that high urinary Na levels or high Na/K ratios (reflecting high Na and low K levels) may be a risk factor for cardiovascular disease and stroke (Averill et al., 2019; Jung et al., 2019). However, the Na/K ratio has been shown to be the superior metric in relation to hypertension when compared to either single Na or K measurements (Stamler et al., 1989).

Here, we found evidence for increased Na/K ratios for both wet- and dry-weight measurements in VaD brain tissue, as well as similar ratio increases in AD from our previous AD metallomic dataset (Xu et al., 2017). Although these measurements were calculated using *post-mortem* brain tissue, if these ratios can be replicated in VaD patient urine samples, for example, then the measurement of Na/K ratios may prove to be informative if used as an additive measure for the identification of at-risk individuals. However, further research is first required to see if such differences are also evident in preclinical VaD.

In addition to maintaining ionic gradients, the Na^+^/K^+^-ATPase also regulates cell volume (Pivovarov et al., 2018). However, unlike previous reports of brain-water content and Na/K levels (Young et al., 1987; Yang et al., 1992), here, we found no evidence for case-control differences in hippocampal-water concentrations (Supplementary Table 3). This implies the maintenance of osmotic equilibrium despite evidence of Na and K ion imbalances in VaD *post-mortem* hippocampal tissue. These findings highlight the possibility of additional mechanisms contributing to the hippocampal-metal dyshomeostasis observed in VaD. However, what these mechanisms might be will require further investigation.

The study of physiological Cu levels in neurodegenerative diseases has gained considerable attention due to its essentiality in the functioning of cytochrome C oxidase (electron transport chain complex IV) and its use as a cofactor for various antioxidant enzymes (Uauy et al., 1998). Previous reports of Cu levels have shown contrasting hippocampal levels in AD (Xu et al., 2017) and Parkinson’s disease dementia (PDD) (Scholefield et al., 2021) on the one hand, and type-2 diabetes (T2D) (Philbert et al. in submission) on the other, whereby AD and PDD exhibited decreased Cu and T2D increased Cu levels. Here, mean Cu levels were moderately higher in VaD hippocampal dry-weight tissue compared to controls, although this between-group difference was just over the 0.05 significance threshold when using non-parametric measurements. However, when parametric analyses were employed, this difference was below the threshold (*p* = 0.0497). In contrast, although of borderline significance, mean Cu levels in wet-weight tissue were probably decreased in the VaD group. This difference between wet- and dry-weight measurements from the same tissue may well reflect the need for a larger sample size as there was a greater degree of wet-weight variability in the control group compared to the VaD group, which could have impacted the results. The notion that an increased sample size would be helpful is further supported by the low statistical power (<80%) observed for both dry- and wet-weight Cu measurements (Supplementary Tables 4, 5). Additional analysis of brain-Cu levels, preferably in a larger multi-regional cohort, will be necessary to better understand its relevance in VaD and to determine whether alterations in brain-Cu levels have a more marked or different association with AD/PDD or with T2D.

As there is little evidence surrounding the minimum recommended sample size for normality tests, which help determine whether the parametric assumptions are met, here, we have interpreted results from both parametric and non-parametric analyses. However, it is important to note that Na and K perturbations were observed using either type of analysis. The only differences observed were increased Cu and Zn (*p* = 0.0497 and *p* = 0.0498, respectively) in wet-weight VaD hippocampal tissue using parametric analysis; the strength of this evidence concerning Cu or Zn levels in the hippocampus of VaD patients is weak and this matter requires further investigation.

As we had previously characterized hippocampal-metal concentrations from T2D and various age-related dementias (AD, PDD, HD) using the same methodology, PCA was conducted to further understand the metallomic differences observed in VaD in relation to these other neurodegenerative diseases. Although PCA is primarily employed in the testing of large datasets to reduce the number of dimensions, such unsupervised methods can also be particularly useful in revealing patterns in small-scale metallomic data. Prior testing using both the Kaiser-Meyer Olkin measure for sampling accuracy and Bartlett’s test for sphericity confirmed the utility of our multi-dementia dataset for PCA. However, no apparent discrimination was visible between all cohorts, including controls. Furthermore, the top loading scores, which represent the contribution of each metal to the PCs, only showed a modest relationship. This finding implies that despite evidence for cohort-specific metal perturbations in each of these diseases, when grouped together, the overall metallomic profile shows no distinguishable pattern. The hippocampus is understood to be a severely affected region in AD, but only a moderately affected region in VaD and T2D, and even less affected in PDD and HD. Therefore, under the assumption that brain-metal perturbations may be greater in more severely affected regions, data from diseases known to undergo less hippocampal atrophy may have had an impact here on PCA cluster separation, although, one would expect greater discrimination between cohorts based on diseases with low vs high hippocampal atrophy. Therefore, it may be necessary to perform a larger multiregional study to further understand the effects of different regions on PCA cluster separation. The absence of inter-group discrimination after PCA may have also been influenced by brain bank variability. In a study that assessed the influence of several covariates on human AD *post-mortem* tissue metal levels; K, Cu, and Se were shown to be significantly affected by differences in brain-bank identity (Scholefield et al., 2020). As the present study used metallomic data from a range of tissue sourced from UK brain banks, as well as brain banks based both in the United States and in New Zealand, it is possible that such variability could have affected the PCA inter-group separation, although to what extent has not yet been determined.

In summary, to our knowledge, this is the first study to utilize ICP-MS to assess the metallomic status of hippocampal VaD *post-mortem* tissue. Taken together, these results present novel evidence for contrasting Na and K perturbations in VaD hippocampal tissue as a likely consequence of Na^+^/K^+^-ATPase dysfunction resulting from defective energy availability. While the idea of Na^+^/K^+^-ATPase dysfunction in VaD has been previously suggested (Adav et al., 2014), the contribution of such perturbations on Na and K levels in brain tissue has not been investigated, until now. Elevations in Na and Na/K ratios may also prove useful in the detection of VaD and/or at-risk individuals, although further research will be necessary to confirm their diagnostic efficacy. Furthermore, in contrast to previously analyzed hippocampal metallomic datasets of dementia-causing neurodegenerative diseases, multivariate analysis identified no distinguishable patterns between cohorts. We propose that future studies should probe the clinical utility of hippocampal-Na levels in VaD, for example, possibly through brain-^23^Na magnetic resonance imaging, to further understand its diagnostic potential in neurodegenerative disease.

## Data Availability Statement

The original contributions presented in the study are included in the article/Supplementary Material, further inquiries can be directed to the corresponding authors.

## Author Contributions

SP designed and performed the experiments, analyzed and interpreted the data, and wrote the manuscript. JX performed the experiments and revised the manuscript. SC performed the experiments and analyzed the data. MS performed the experiments, analyzed the data, and revised the manuscript. RU designed the experiments, interpreted the data, and revised the manuscript. GC designed the experiments, interpreted the data, revised the manuscript, and bears overall responsibility for the integrity of this manuscript and of the study. All authors contributed to the article and approved the submitted version.

## Conflict of Interest

The authors declare that the research was conducted in the absence of any commercial or financial relationships that could be construed as a potential conflict of interest.

## Publisher’s Note

All claims expressed in this article are solely those of the authors and do not necessarily represent those of their affiliated organizations, or those of the publisher, the editors and the reviewers. Any product that may be evaluated in this article, or claim that may be made by its manufacturer, is not guaranteed or endorsed by the publisher.
