# Supplementary material for: Contrasting Sodium and Potassium Perturbations in the Hippocampus Indicate Potential Na+/K+-ATPase Dysfunction in Vascular Dementia
Source: Front Aging Neurosci. 2022 Jan 28;14:822787. doi: 10.3389/fnagi.2022.822787 (PMC8832097; doi:10.3389/fnagi.2022.822787)
Supplement: Supplementary file 1 [file Data_Sheet_1.pdf]

Supplementary Material

Supplementary Tables

Supplementary Table 1. Individual patient characteristics

Code	Class	Age at death	Sex	Brain wt (g)	PMD (h)	Cause of death*
72	Control	72	M	1300	42	Ruptured abdominal aortic aneurysm, ischaemic heart disease
122	Control	82	M	1480	30	Congestive cardiac failure, ischaemic heart disease, UTI
781	Control	87	M	1364	24	Acute renal failure, myeloma
930	Control	94	F	1218	29.5	Oesophageal carcinoma, ischemic heart disease
943	Control	70	F	1032	33.25	Carcinomatosis, metastatic squamous cell carcinoma of larynx
948	Control	82	F	1135	36	Carcinomatosis, ovarian/peritoneal carcinoma
957	Control	86	M	1345	44.25	Infective exacerbation of COPD
1067	Control	69	F	1121	38.25	Metastatic pancreatic cancer
1083	Control	94	F	1166	43.25	Pneumonia, frailty of old age
1092	Control	86	F	1288	36.25	Mucinous adenocarcinoma of appendix
32	VaD	84	F	1230	20	Not specified†
92	VaD	72	M	1460	41	Not specified†
131	VaD	86	F	1060	28	Not specified†
170	VaD	90	F	1150	31	Not specified†
232	VaD	89	M	1330	30	Pulmonary embolism, ischaemic heart disease, old age
347	VaD	76	M	1094	40	Not specified†
787	VaD	78	F	1161	54	Vascular dementia
931	VaD	76	F	1293	50	Urosepsis
1008	VaD	87	M	1160	34.25	Old age, vascular dementia, leg ulcer, type-2 diabetes
1105	VaD	98	F	1169	22.75	Vascular dementia

†Those patients in the VaD Class whose cause of death was not specified in the database had VaD as determined by post-mortem examination but not specified at the time of writing as a cause of death in the SWDBB database. Abbreviations: COPD: Chronic obstructive pulmonary disease; PMD: *Post-mortem* delay; UTI; Urinary tract infection; wt: Weight. *Cause of death as specified by the SWDBB database.

Supplementary Table 2. ICP-MS calibration standard solutions

Internal standard solution	Solution	
100 μg/L internal standard	10 mL 2% nitric acid solution	100 μL environmental calibration standard mixture
50 μg/L internal standard	15 mL 2% nitric acid solution	75 μL environmental calibration standard mixture
20 μg/L internal standard	4 mL 2% nitric acid solution	1 mL 100 μg/L solution
10 μg/L internal standard	9 mL 2% nitric acid solution	1 mL 100 μg/L solution
5 μg/L internal standard	9 mL 2% nitric acid solution	1 mL 50 μg/L solution
2 μg/L internal standard	4 mL 2% nitric acid solution	1 mL 10 μg/L solution
1 μg/L internal standard	9 mL 2% nitric acid solution	1 mL 10 μg/L solution
0.5 μg/L internal standard	9 mL 2% nitric acid solution	1 mL 5 μg/L solution
Blank	10 mL 2% nitric acid solution	-

Supplementary Table 3. Individual sample wet-weight/dry-weight hippocampal tissue ratios

Sample ID	Class	Wet weight	Dry weight	Tissue (%)	Water (%)	Ratio
72	Control	46.7	9.6	20.6	79.4	4.86
122	Control	52.5	9.6	18.3	81.7	5.47
781	Control	46.3	8.3	17.9	82.1	5.58
930	Control	51.8	11	21.2	78.8	4.71
943	Control	53.7	9.4	17.5	82.5	5.71
948	Control	47.9	8.4	17.5	82.5	5.70
957	Control	53.9	9.5	17.6	82.4	5.67
1067	Control	53.1	9.4	17.7	82.3	5.65
1083	Control	48.1	7	14.6	85.4	6.87
1092	Control	46.3	7.1	15.3	84.7	6.52
32	VaD	46	7.3	15.9	84.1	6.30
92	VaD	54.6	11	20.1	79.9	4.96
131	VaD	47.3	10.1	21.4	78.6	4.68
170	VaD	50.6	7.5	14.8	85.2	6.75
232	VaD	45.7	8.1	17.7	82.3	5.64
347	VaD	52.5	10.2	19.4	80.6	5.15
787	VaD	48.8	6.7	13.7	86.3	7.28
931	VaD	49.7	7.2	14.5	85.5	6.90
1008	VaD	51.6	8.8	17.1	82.9	5.86
1105	VaD	50	8.5	17.0	83.0	5.88
Av. Control		50.03	8.93	17.83	82.17	5.68
Av. VaD		49.68	8.54	17.16	82.84	5.94

No statistically significant case-control differences were observed for hippocampal tissue ratios.

Supplementary Table 4. Dry-weight power analysis, effect size, and sample size estimates

Element	Power ($p < 0.05$)	Required sample size ($p < 0.05$)	Effect size
Na	0.652	46	1.111
Mg	0.187	208	0.504
K	0.144	298	0.420
Ca	0.207	182	0.538
Mn	0.081	890	0.242
Fe	0.072	1246	0.205
Cu	0.512	62	0.941
Zn	0.517	62	0.946
Se	0.211	178	0.545

Supplementary Table 5. Wet-weight power analysis, effect size, and sample size estimates

Element	Power ($p < 0.05$)	Required sample size ($p < 0.05$)	Effect size
Na	0.936	22	1.648
Mg	0.090	692	0.275
K	0.797	32	1.321
Ca	0.260	138	0.620
Mn	0.070	1362	0.196
Fe	0.215	174	0.551
Cu	0.097	584	0.299
Zn	0.051	46290	0.034
Se	0.081	894	0.244

Supplementary Table 6. PCA loading scores for the multiple age-related dementia dry-weight metallomic data

Element	PC1	PC2
Na	-0.359	0.348
Mg	**-0.443**	-0.101
K	-0.352	**-0.428**
Ca	-0.217	**0.470**
Mn	-0.365	0.352
Fe	-0.293	0.128
Cu	-0.310	-0.426
Zn	**-0.398**	0.001
Se	-0.174	-0.378

The loadings indicate the correlation coefficients between elements and components. The top two loadings with the most extreme value (positive or negative) for each principal component are represented in bold. Abbreviation: PC: Principal component.

Supplementary Table 7. Dry-weight individual patient metal concentrations

**Hippocampus**		^{23}Na	^{24}Mg	^{39}K	^{44}Ca	^{55}Mn	^{56}Fe	^{63}Cu	^{66}Zn	^{78}Se
Sample ID	Class	mmol/kg	mmol/kg	mmol/kg	mmol/kg	µmol/kg	mmol/kg	µmol/kg	µmol/kg	µmol/kg
32-B1	VaD	704	32	289	18.2	35	5.45	322	1395	10.96
92-B3	VaD	492	26	251	8.3	25	3.60	281	1331	9.33
131-B5	VaD	439	26	297	11.9	23	4.72	235	1111	31.01
170-B6	VaD	672	33	396	12.7	27	4.03	359	1728	10.94
232-B7	VaD	460	29	333	8.4	24	3.26	397	1921	12.48
347-B8	VaD	482	27	286	8.0	22	3.62	288	1524	9.23
787-B10	VaD	967	28	280	15.1	29	3.32	348	1809	9.42
931-B12	VaD	681	31	280	52.5	52	4.29	306	1720	9.35
1008-B16	VaD	587	29	275	15.7	25	5.22	347	1331	8.89
1105-B20	VaD	603	31	357	12.9	32	5.03	457	1266	11.12
72-B2	Control	394	26	317	13.0	23	3.83	271	906	9.31
122-B4	Control	430	29	302	8.1	27	5.85	299	1529	9.57
781-B9	Control	491	27	276	11.9	23	3.69	204	1148	9.14
930-B11	Control	336	27	308	7.8	26	3.47	256	967	8.62
943-B13	Control	505	28	336	11.0	32	3.52	267	1486	8.87
948-B14	Control	446	28	366	10.8	26	4.33	296	1326	10.22
957-B15	Control	518	26	266	8.5	28	3.63	264	1220	11.37
1067-B17	Control	380	31	402	12.1	24	4.93	348	1463	10.47
1083-B18	Control	658	32	340	18.2	38	4.82	376	1436	11.29
1092-B19	Control	548	27	296	11.1	28	2.90	210	1297	9.24
	**Av. VaD**	609	29	304	16.4	29	4.25	334	1514	12.27
	**Av. Control**	471	28	321	11.3	28	4.10	279	1278	9.81
	**Mann-Whitney U test**	**0.0355**	0.248	0.280	0.280	0.853	0.796	0.052	0.089	0.315
	**Welch's t-test**	**0.033**	0.267	0.396	0.258	0.541	0.683	0.052	**0.045**	0.277

Supplementary Table 8. Wet-weight individual patient metal concentrations

**Hippocampus**		^{23}Na	^{24}Mg	^{39}K	^{44}Ca	^{55}Mn	^{56}Fe	^{63}Cu	^{66}Zn	^{78}Se
Sample ID	mmol/kg	mmol/kg	mmol/kg	mmol/kg	µmol/kg	mmol/kg	µmol/kg	µmol/kg	µmol/kg	µmol/kg
32-A1	VaD	107	4.54	41	3.84	4.92	0.711	39	187	1.62
92-A3	VaD	89	4.43	44	1.67	4.38	0.615	40	206	1.63
131-A5	VaD	88	5.29	57	2.76	5.05	1.077	46	240	7.43
170-A6	VaD	91	4.89	59	1.39	4.32	0.652	65	267	2.03
232-A7	VaD	76	4.65	53	2.46	3.80	0.639	53	262	1.93
347-A8	VaD	92	4.61	46	1.76	5.28	0.521	64	278	1.62
787-A10	VaD	128	3.97	37	2.12	4.01	0.374	44	280	1.51
931-A12	VaD	95	5.75	40	31.17	16.81	0.750	42	285	1.39
1008-A16	VaD	105	4.42	43	1.99	4.58	0.540	53	223	1.34
1105-A20	VaD	99	4.59	49	1.92	5.27	0.535	45	208	1.67
72-A2	Control	80	5.25	64	1.51	5.97	0.649	89	214	2.02
122-A4	Control	81	5.02	55	1.98	4.85	1.091	56	256	1.88
781-A9	Control	82	4.95	52	1.85	4.20	0.718	65	253	1.90
930-A11	Control	71	4.79	60	2.03	6.92	0.728	80	285	1.76
943-A13	Control	72	4.31	52	1.77	4.35	0.700	39	241	1.59
948-A14	Control	76	4.47	60	1.76	4.41	0.749	44	199	1.59
957-A15	Control	88	4.71	50	1.43	4.41	0.637	48	226	2.11
1067-A17	Control	61	5.48	69	1.64	4.46	0.780	55	250	1.64
1083-A18	Control	92	4.65	49	3.32	5.09	0.701	45	209	1.42
1092-A19	Control	81	4.53	50	1.71	5.05	0.471	30	282	1.51
	**Av. VaD**	97	4.71	47	5.11	5.84	0.641	49	244	2.22
	**Av. Control**	78	4.82	56	1.90	4.97	0.722	55	242	1.74
	**Mann-Whitney test U**	**0.0015**	0.436	**0.012**	0.143	0.796	0.166	0.579	>0.9999	0.739
	**Welch's t-test**	**0.0027**	0.597	**0.010**	0.298	0.506	0.306	0.386	0.889	0.441

Supplementary Table 9. Log-transformed dry-weight individual patient metal concentrations

Hippocampus		^{23}Na	^{24}Mg	^{39}K	^{44}Ca	^{55}Mn	^{56}Fe	^{63}Cu	^{66}Zn	^{78}Se
Sample ID	mmol/kg	mmol/kg	mmol/kg	µmol/kg	mmol/kg	µmol/kg	µmol/kg	µmol/kg	µmol/kg	umol/kg
32-B1	VaD	2.85	1.50	2.46	1.26	1.54	0.736	2.51	3.14	1.040
92-B3	VaD	2.69	1.42	2.40	0.92	1.41	0.556	2.45	3.12	0.970
131-B5	VaD	2.64	1.42	2.47	1.08	1.37	0.674	2.37	3.05	1.491
170-B6	VaD	2.83	1.51	2.60	1.10	1.43	0.605	2.56	3.24	1.039
232-B7	VaD	2.66	1.46	2.52	0.92	1.39	0.513	2.60	3.28	1.096
347-B8	VaD	2.68	1.43	2.46	0.90	1.35	0.559	2.46	3.18	0.965
787-B10	VaD	2.99	1.45	2.45	1.18	1.46	0.521	2.54	3.26	0.974
931-B12	VaD	2.83	1.49	2.45	1.72	1.71	0.632	2.49	3.24	0.971
1008-B16	VaD	2.77	1.46	2.44	1.20	1.39	0.718	2.54	3.12	0.949
1105-B20	VaD	2.78	1.49	2.55	1.11	1.51	0.702	2.66	3.10	1.046
72-B2	Control	2.60	1.42	2.50	1.12	1.36	0.584	2.43	2.96	0.969
122-B4	Control	2.63	1.46	2.48	0.91	1.43	0.767	2.48	3.18	0.981
781-B9	Control	2.69	1.43	2.44	1.08	1.36	0.567	2.31	3.06	0.961
930-B11	Control	2.53	1.43	2.49	0.89	1.42	0.540	2.41	2.99	0.936
943-B13	Control	2.70	1.44	2.53	1.04	1.50	0.546	2.43	3.17	0.948
948-B14	Control	2.65	1.45	2.56	1.03	1.42	0.636	2.47	3.12	1.010
957-B15	Control	2.71	1.42	2.43	0.93	1.44	0.560	2.42	3.09	1.056
1067-B17	Control	2.58	1.49	2.60	1.08	1.37	0.693	2.54	3.17	1.020
1083-B18	Control	2.82	1.50	2.53	1.26	1.58	0.683	2.57	3.16	1.053
1092-B19	Control	2.74	1.42	2.47	1.05	1.45	0.463	2.32	3.11	0.966
	**Av. VaD**	2.77	1.46	2.48	1.14	1.45	0.622	2.52	3.17	1.054
	**Av. Control**	2.67	1.45	2.50	1.04	1.43	0.604	2.44	3.10	0.990
	**Welch's t test**	**0.024**	0.268	0.367	0.249	0.606	0.652	**0.0497**	**0.0498**	0.248

Supplementary Table 10. Log-transformed wet-weight individual patient metal concentrations

Hippocampus		^{23}Na	^{24}Mg	^{39}K	^{44}Ca	^{55}Mn	^{56}Fe	^{63}Cu	^{66}Zn	^{78}Se
Sample ID	mmol/kg	mmol/kg	mmol/kg	µmol/kg	mmol/kg	µmol/kg	µmol/kg	µmol/kg	µmol/kg	umol/kg
32-A1	VaD	2.03	0.657	1.61	0.584	0.692	-0.148	1.59	2.27	0.209
92-A3	VaD	1.95	0.647	1.65	0.222	0.641	-0.211	1.60	2.31	0.212
131-A5	VaD	1.94	0.722	1.76	0.440	0.703	0.032	1.66	2.38	0.871
170-A6	VaD	1.96	0.689	1.77	0.144	0.635	-0.186	1.81	2.43	0.306
232-A7	VaD	1.88	0.667	1.73	0.391	0.580	-0.195	1.72	2.42	0.285
347-A8	VaD	1.97	0.664	1.66	0.245	0.723	-0.283	1.80	2.44	0.210
787-A10	VaD	2.11	0.599	1.57	0.327	0.603	-0.428	1.64	2.45	0.178
931-A12	VaD	1.98	0.759	1.60	1.494	1.226	-0.125	1.62	2.45	0.143
1008-A16	VaD	2.02	0.645	1.63	0.300	0.661	-0.268	1.73	2.35	0.128
1105-A20	VaD	2.00	0.662	1.69	0.284	0.722	-0.271	1.66	2.32	0.222
72-A2	Control	1.90	0.720	1.81	0.177	0.776	-0.188	1.95	2.33	0.305
122-A4	Control	1.91	0.701	1.74	0.296	0.686	0.038	1.74	2.41	0.274
781-A9	Control	1.91	0.695	1.71	0.266	0.624	-0.144	1.81	2.40	0.279
930-A11	Control	1.85	0.680	1.78	0.308	0.840	-0.138	1.90	2.46	0.246
943-A13	Control	1.86	0.635	1.71	0.249	0.638	-0.155	1.59	2.38	0.201
948-A14	Control	1.88	0.651	1.78	0.244	0.644	-0.126	1.64	2.30	0.203
957-A15	Control	1.95	0.673	1.70	0.156	0.645	-0.196	1.68	2.35	0.325
1067-A17	Control	1.78	0.739	1.84	0.216	0.649	-0.108	1.74	2.40	0.215
1083-A18	Control	1.96	0.667	1.69	0.521	0.707	-0.154	1.66	2.32	0.152
1092-A19	Control	1.91	0.656	1.70	0.234	0.704	-0.327	1.47	2.45	0.178
	**Av. VaD**	1.98	0.671	1.67	0.443	0.719	-0.208	1.68	2.38	0.276
	**Av. Control**	1.89	0.682	1.75	0.267	0.691	-0.150	1.72	2.38	0.238
	**Welch's t-test**	**0.0018**	0.552	**0.0089**	0.195	0.670	0.235	0.519	0.932	0.597

Supplementary Figures

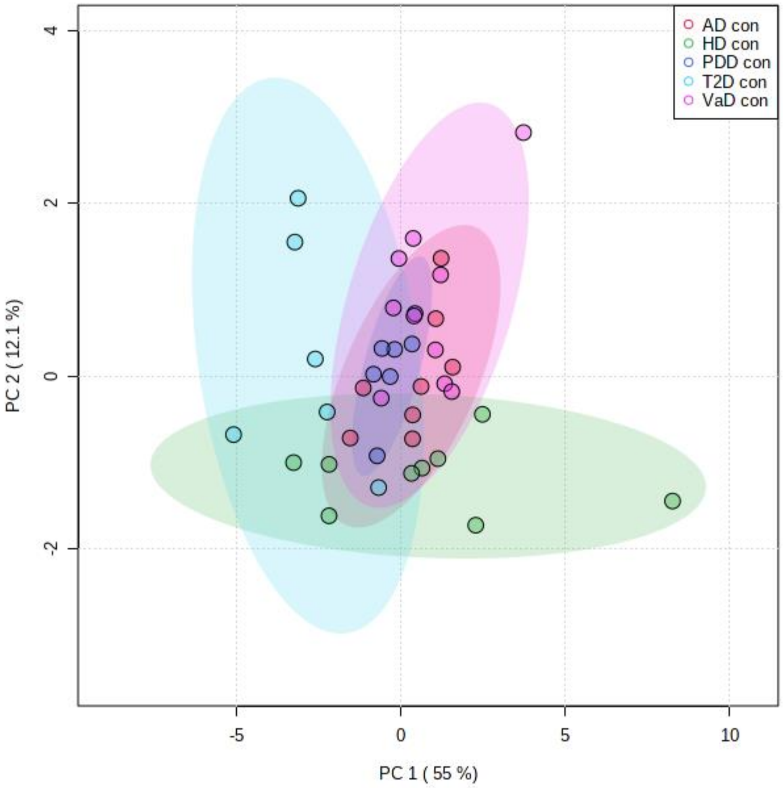

Supplementary Figure 1. Two dimensional PCA plot for human dry-weight hippocampal *post-mortem* tissue from multiple control cohorts. Data represent a PCA plot using ICP-MS-metallomic data from VaD con (n = 10; pink), AD con (n = 8; red), HD con (n = 9; green), PDD con (n = 7; dark blue), T2D con (n = 6; light blue) dry-weight hippocampal *post-mortem* tissue. The coloured ellipses around each cohort signify 95% confidence regions. The first (Dim1) and second (Dim2) principal components contribute to 55% and 12.1% of the total variance, respectively. No visible separation was apparent between all cohorts. Abbreviations: AD: Alzheimer's disease; Con: Control; ICP-MS: Inductively coupled plasma-mass spectrometry; PDD: Parkinson's disease dementia; T2D: Type-2 diabetes; VaD: Vascular dementia.

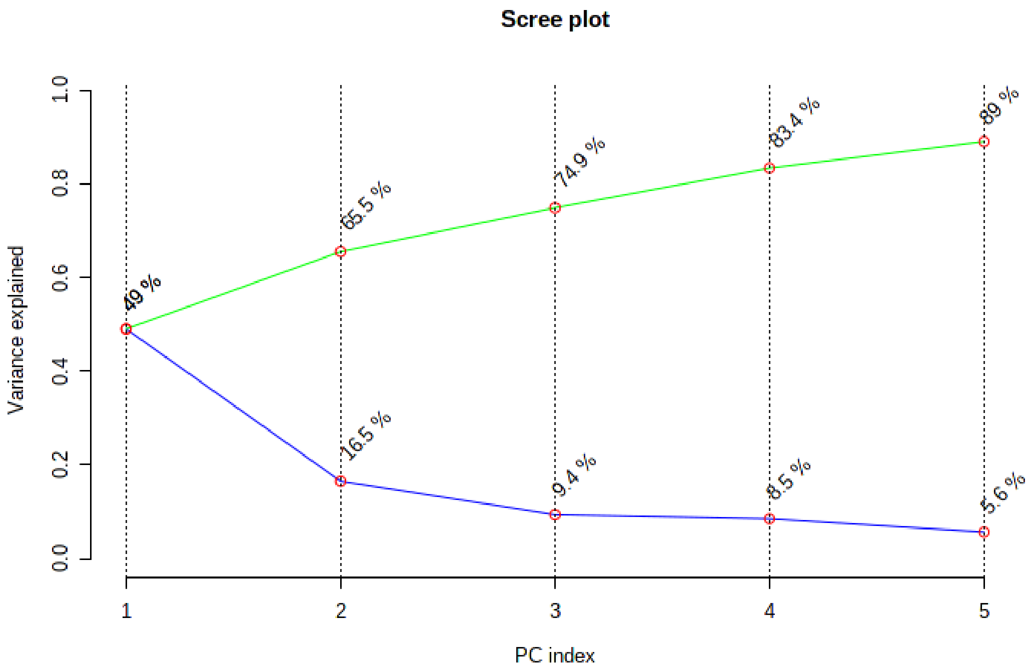

Supplementary Figure 2. Scree plot for human dry-weight hippocampal *post-mortem* tissue from multiple age-related demented diseases and T2D. Data shown represents the variance explained for the first five dimensions (principal components) within the dry-weight multiple dementia dataset.

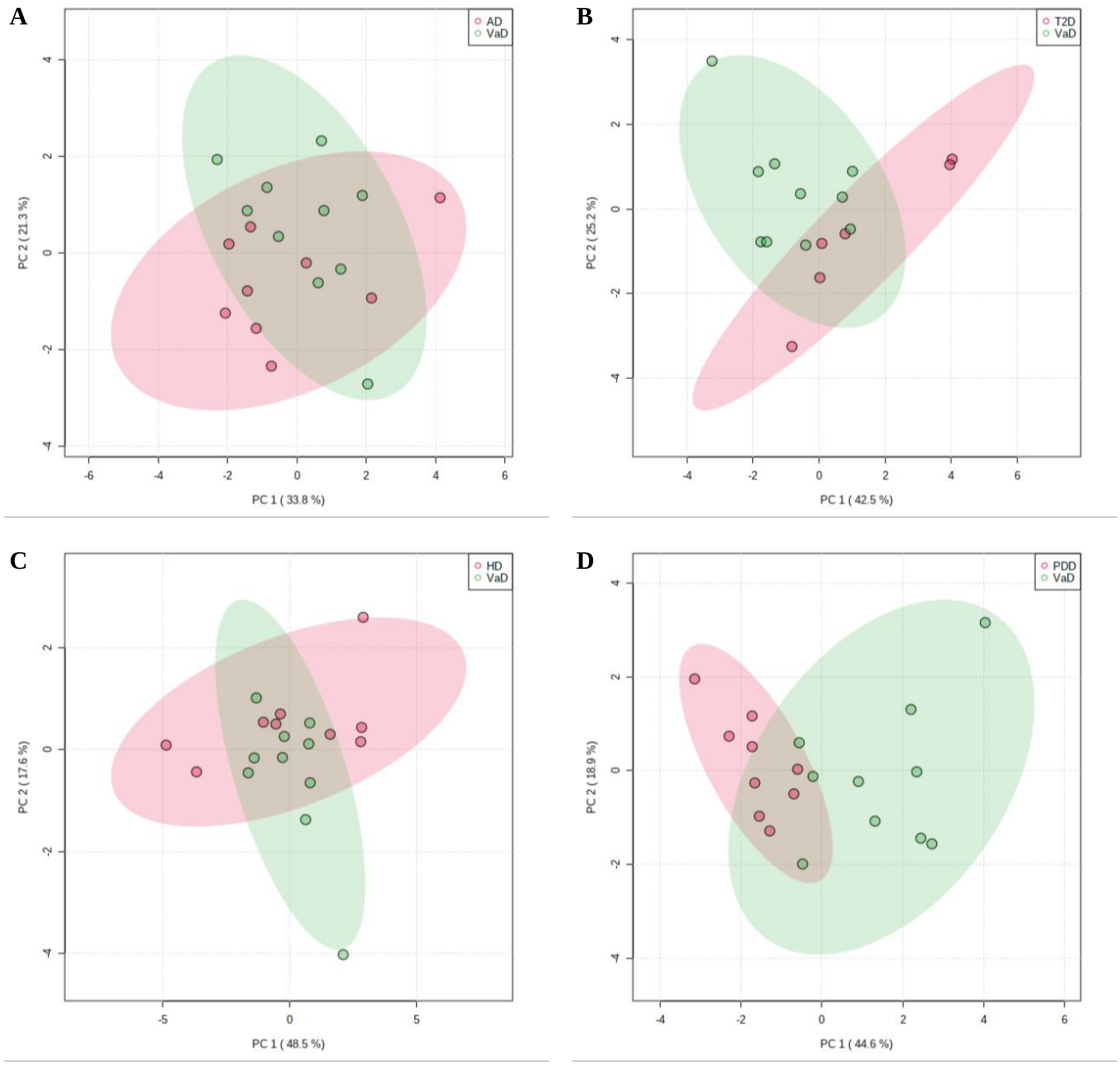

Supplementary Figure 3. Two dimensional PCA pairwise disease comparisons for human dry-weight hippocampal *post-mortem* tissue between VaD and other dementia-causing diseases. Data represent pairwise PCA disease comparison plots using ICP-MS metallomic data. Comparisons are: A) VaD vs AD; B) VaD vs T2D; C) VaD vs HD; and D) VaD vs PDD. No visible separation was apparent across all disease comparisons. Abbreviations: AD: Alzheimer's disease; Con: Control; ICP-MS: Inductively coupled plasma-mass spectrometry; PDD: Parkinson's disease dementia; T2D: Type-2 diabetes; VaD: Vascular dementia.
